# Editorial: The Global Methamphetamine Problem: Approaches to Elucidate the Neurobiology, Epidemiology, and Therapeutic Effectiveness

**DOI:** 10.3389/fpsyt.2020.00850

**Published:** 2020-08-19

**Authors:** Milky Kohno, Christian Beste, Maximilian Pilhatsch

**Affiliations:** ^1^ Department of Psychiatry, Oregon Health and Science University, Portland, OR, United States; ^2^ Department of Behavioral Neuroscience, Oregon Health and Science University, Portland, OR, United States; ^3^ Research and Development Service, Veterans Affairs Portland Health Care System, Portland, OR, United States; ^4^ Methamphetamine Abuse Research Center, Oregon Health and Science University and Veterans Affairs Portland Health Care System, Portland, OR, United States; ^5^ Cognitive Neurophysiology, Department of Child and Adolescent Psychiatry, Faculty of Medicine, TU Dresden, Dresden, Germany; ^6^ Department of Psychiatry and Psychotherapy, Technische Universität Dresden, Dresden, Germany; ^7^ Department of Psychiatry and Psychotherapy, Elblandklinikum Radebeul, Radebeul, Germany

**Keywords:** methamphetamine, crystal meth, psychotherapy, mechanisms, transferability, Europe, global

Methamphetamine-use disorder (MUD) is a global problem and is of great public health concern. The rapid increase in methamphetamine (MA) use in Europe, particularly in young adults has led to a significant medical shortfall in many regions. MUD is a particularly difficult addiction to treat, in part, because of the psychiatric comorbidities and the effects of MA on the neurobiological mechanisms that affect higher-order cognitive functions relevant for adaptive behavior and successful completion of treatment programs. Moreover, little is known about the risk factors and susceptibility of MUD or the trajectories in neurocognitive and neurobiological deficits and treatment response. This special issue on the global methamphetamine problem, therefore, focuses on MA use as a multi-faceted construct that needs to be evaluated in the context of risk factors, neurobiology, therapeutic approaches, and comorbidities that likely interact with treatment outcomes.

We start with the cognitive dysfunctions associated with MUD, as these deficits have lasting impact on daily life behavior and treatment outcomes. The systematic review presented by May et al. highlights the abnormalities in emotion regulation, goal-directed decision making, and responses to negative reinforcement in MUD. This review provides a comprehensive evaluation of MA-associated cognitive deficits, which have been considered in the following papers examining abstinence and treatment. For example, in Bernhardt et al. patients with MUD show improvements in sustained attention but no change in impulsive choices after 3 months of abstinence. Similarly, Bensmann et al. report that some tests of executive function are impaired, while others normalized after abstinence. These differences in executive function have significant implications for treatment. The study presented by Lake et al. presents evidence that individual variability in the aversion to losses and in the predilection for large and immediate rewards is a factor in successful contingency management treatment outcomes. In line with these results, naltrexone-induced changes in large-scale brain networks that are important for many of the deficits in executive function seen in MUD is associated with MA abstinence and addiction severity (Kohno et al.). Together, these studies show the importance of interventions to consider cognitive control and decision-making deficits as a factor in order to enhance the effectiveness of different treatment approaches.

Cognitive deficits and maladaptive decision-making associated with MUD also has implications on public health at large, which is highlighted in a paper by Schecke et al. that reports that MA use in sexual settings is related to higher rates of HIV. In addition to the public health concern, an increase in mental health disorders are associated with MA use in sexual settings, which underscores the importance of identifying psychiatric comorbidities when treating MUD. For example, comorbid substance use disorders influence the trajectory of MUD recovery, where the presence of a dual diagnosis is associated with greater occurrence of relapse, death, or incarceration (Tan et al.). Similarly, incarceration rates in MUD interacted with levels of psychopathy and corticostriatal brain connectivity (Hoffmann et al.). As this brain network is important for executive function and cognitive control, these results are in line with the study presented by Arunogiri et al. that show impairments of emotion recognition and impulsive choice in MUD and the additional presence of psychotic symptoms potentiating these effects.

Treatments that limit MA use through improvements in decision-making skills or modification of neural networks are imperative, as a study that evaluates the first German-language therapy manual for specific short-term treatment of MUD (Petzold et al.) shows that shorter periods of MA use is a primary predictor for positive treatment responses. An innovative study protocol to limit MA use has also been proposed, which will examine the efficacy of retrieval-extinction training combined with virtual reality to reduce cue-evoked responses in MUD (Liu et al.). Another important consideration in reducing MA use is genetic risk factors. A preclinical study showing that Homer2 expression regulates the rewarding/reinforcing properties of MA highlights the need to identify genetic susceptibility for MUD to develop tailored models for prevention and treatment (Brown et al.).

Overall, the selection of these studies highlights the complex dynamics of MUD and the need for interdisciplinary research. Extending the results from these published articles would be of great value in identifying the interactions between psychosocial, genetic, neural and behavioral markers that are associated with stimulant use and has the potential to advance therapeutic strategies for addiction.

## Author Contributions

MK, CB, and MP wrote the manuscript. All authors contributed to the article and approved the submitted version.

## Funding

This work was funded by the MeDDrive program of the Technische Universität Dresden (MeDDrive grant #60.401), the Deutsche Forschungsgemeinschaft (DFG, German Research Foundation) – Project-ID 402170461 – TRR 265 (1) and the Department of Veterans Affairs Clinical Sciences Research and Development Career Development Award IK2CX001790, Oregon Health & Science University Collins Medical Trust Award APSYC0249, Medical Research Foundation of Oregon APSYC0250, and the Center for Women’s Health - Circle of Giving APSYC0287.

The funding bodies had no further role in the conceptualization of the study, the collection and analysis of data, the preparation of the manuscript or the decision to publish.

## Conflict of Interest

The authors declare that the research was conducted in the absence of any commercial or financial relationships that could be construed as a potential conflict of interest.
